# Building capacity to encourage research reproducibility and #MakeResearchTrue

**DOI:** 10.5195/jmla.2018.273

**Published:** 2018-01-02

**Authors:** Melissa L. Rethlefsen, Mellanye J. Lackey, Shirley Zhao

## Abstract

**Background:**

Research into study replication and reporting has led to wide concern about a reproducibility crisis. Reproducibility is coming to the attention of major grant funders, including the National Institutes of Health, which launched new grant application instructions regarding rigor and reproducibility in 2015.

**Study Purpose:**

In this case study, the authors present one library’s work to help increase awareness of reproducibility and to build capacity for our institution to improve reproducibility of ongoing and future research.

**Case Presentation:**

Library faculty partnered with campus research leaders to create a daylong conference on research reproducibility, followed by a post-conference day with workshops and an additional seminar. Attendees came from nearly all schools and colleges on campus, as well as from other institutions, nationally and internationally. Feedback on the conference was positive, leading to efforts to sustain the momentum achieved at the conference. New networking and educational opportunities are in development.

**Discussion:**

Libraries are uniquely positioned to lead educational and capacity-building efforts on campus around research reproducibility. Costs are high and partnerships are required, but such efforts can lead to positive change institution-wide.

## INTRODUCTION

In 2005, John Ioannidis published one of the most influential articles of recent decades in *PLOS Medicine* [1]. “Why Most Published Research Findings Are False,” Ioannidis’ paper, has been viewed over 2 million times and cited more than 2,500 times. His premise was confrontational and highly controversial, but it spawned a renewed interest in research integrity, especially research reproducibility.

Urgency around these issues increased dramatically in 2012, when Begley and Ellis published a report in *Nature* about Amgen scientists’ unsuccessful attempt to replicate findings from 47 of 53 preclinical studies [2]. This remarkable failure to reproduce 89% of the studies that they attempted to replicate resounded through the biomedical community and led directly to increased attention to the quality of biomedical research. Additional mass replication studies, including the Open Science Collaboration’s Reproducibility Project [3] in psychology and their preliminary results in cancer biology [4], have shown slightly less poor replicability but still enough to cause concern of a looming “reproducibility crisis.” Reacting to these and other studies, the National Institutes of Health (NIH) released new grant application instructions and reviewer criteria focusing on research rigor and reproducibility on October 9, 2015 [5, 6].

While there is no single definition of research reproducibility that everyone agrees on, Goodman et al. have recently proposed a unifying “lexicon” for research reproducibility [7]. They posit that reproducibility issues fall into three major categories: methods reproducibility, results reproducibility, and inferential reproducibility. They further elaborate that robustness and generalizability are separate components of reproducibility [7].

Others address reproducibility more from perceived specific causes of irreproducibility, such as poor study design and analysis, bad reference materials and reagents (particularly misidentified cell lines), and poor description of methods and protocols. The NIH emphasizes several of these concepts in their guidance, especially aspects of study design like randomization, blinding, and addressing of biological variables like sex; authentication of biological and chemical resources; and establishment of the scientific premise for the study through review of previous work [5].

What makes a study reproducible is a combination of dozens of factors, each of which—when not designed, executed, or reported accurately—can play a role in making a study irreproducible. It can be mundane, such as not being able to reproduce experimental results because one group shook while another group stirred incubating tissue as part of an otherwise identical study methodology [8], or major, such as the massive issue of the thousands of mislabeled cell lines that can cause researchers who think they are investigating one species or type of cancer to really have published results on something completely different—thus rendering their results totally useless [9].

In addition, outcomes switching [10] and *p*-value hacking [11] are rampant in biomedical literature; both techniques are used to produce results that seem to be more impressive (and are thus considered more publishable) and to fit the results that the investigators expected. Many efforts have been undertaken to improve reproducibility, such as journals removing word limits from methods sections or publishing full methods online [12], creating reporting guidelines to help researchers report their work fully and transparently [13], establishing ClinicalTrials.gov to show original trial protocols (and making it easy to spot how researchers change their outcomes and findings for publication) [14], developing international guidance for cell line authentication [15], and even forbidding the use of *p*-values [16]. Despite these efforts, reproducibility remains a major concern for all areas of biomedicine and scientific inquiry.

## STUDY PURPOSE

Research reproducibility is a major concern for researchers, administrators, and the public [17–20]. Solving the reproducibility crisis is a multipronged, long-term goal that requires governmental, institutional, and individual buy-in [21]. In this case study, the authors present one library’s work to help increase awareness of research reproducibility and to build capacity for our institution to improve reproducibility of ongoing and future research.

## CASE PRESENTATION

On October 15, 2015, knowing of a mutual passion related to this topic, the Spencer S. Eccles Health Sciences Library’s (EHSL’s) deputy director approached the vice president for research (VPR) at the University of Utah with a proposal to jointly sponsor and plan a daylong conference on research reproducibility on November 14, 2016. To assist in planning the conference, the VPR assembled a group of campus faculty who were interested in the topic. These faculty helped generate ideas for speakers and gave feedback throughout the year leading up to the conference. At EHSL, the deputy director recruited three additional library faculty to assist in planning the logistics and programming for the conference.

Additional feedback was sought from the research deans across campus. Funding was secured from the VPR office as well as library endowment funds for InfoFair, an annual EHSL-sponsored conference; the Clifford C. Snyder and Mary Snyder Lectureship; and the Priscilla M. Mayden Lecture. The VPR and the associate VPR strongly recommended focusing the conference around a tangible concept and suggested an institutional-level focus. The article, “How to Make More Published Research True,” also by Ioannidis [21], offered a framework for the conference.

After determining our conference theme and framework, we drafted an agenda. We included slots for three keynote speakers, including two who would serve as our named lecturers; a panel and roundtable discussion with local experts; a panel with local journal editors; a panel with experts from federal agencies who were interested in or required reproducible research; a poster session; and opening and closing remarks. We also invited the Center for Open Science to offer two on-site post-conference workshops on using the Open Science Framework [22]. Funding also enabled us to offer breakfast, lunch, snacks, and free registration for attendees.

Each library faculty member was assigned a particular area of emphasis: One solicited keynote speakers and federal panelists, and worked with the Center for Open Science; one handled the logistics surrounding event, including arranging for continuing medical education (CME) credit for the conference and post-conference workshops; one solicited speakers for the local panels and created the post-conference evaluation tool; and one built the website [23], accompanying LibGuide [24], and registration system, and managed the poster session.

**Figure 1:**
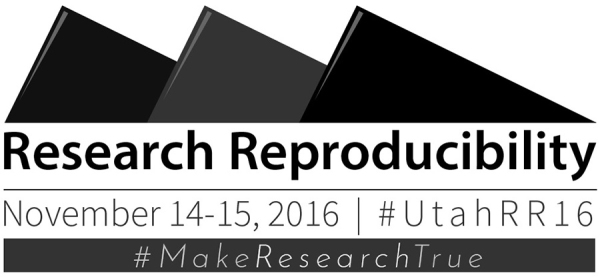
Research Reproducibility logo

We used an internal graphic design expert to design a logo for the conference (Figure 1), and we established two hashtags for the event to use as part of the logo and advertising. The hashtag #UtahRR16 was created specifically to advertise the conference and was the primary hashtag during the conference. The other hashtag, #MakeResearchTrue, was used both for promoting the conference and for sharing reproducibility-related resources on the EHSL Twitter account (@EHSLibrary). All team members were responsible for helping to advertise the conference via social media, university mailing lists, external mailing lists, in-person promotion at meetings and events, and paper flyers.

The final line-up of speakers included international, national, and local experts. We were fortunate to have John Ioannidis (co-director, Meta-Research Innovation Center, Stanford University) as the Snyder lecturer; Hilda Bastian (editor, PubMed Health and PubMed Commons, National Library of Medicine) as the Mayden lecturer; and David Moher (Centre for Journalology, Ottawa Hospital Research Institute) as our featured speaker. These internationally known experts were joined by Kathryn Partin (director, Office of Research Integrity, US Department of Health & Human Services) and Lisa Meier McShane (chief, Biostatistics, Biometric Research Program, US National Cancer Institute), who offered prepared remarks and contributed to a panel session moderated by VPR Emeritus Thomas Parks.

The panel of local journal editors included John Carey (*American Journal of Medical Genetics, Parts A & C*), Julie Fritz (*Journal of Orthopedic and Sports Physical Therapy*), David Grainger (*Biomaterials*), and Patricia Morton (*Journal of Professional Nursing*). The local experts panel included Thomas Cheatham (director, Research Computing and Center for High Performance Computing), J. Michael Dean (co-principal investigator, National Center for Advancing Translational Sciences Trial Innovation Center), Tom Greene (director, Study Design and Biostatistics Center, Center for Clinical and Translational Science [CCTS]), Mellanye J. Lackey (Systematic Review Core, CCTS), and Bernie LaSalle (Biomedical Informatics, CCTS). Both panels were moderated by Melissa L. Rethlefsen, AHIP, who also emceed the conference. Opening remarks were given by Andrew Weyrich, VPR.

In addition to the speakers and panelists, participants were invited to submit posters for a lunchtime poster session. The call for posters went out in May 2016, and acceptances were sent out in August 2016. The call for submissions asked for posters that addressed one or more of the twelve suggestions for making “published research more true” as identified in Ioannidis’s article. A total of twenty-six posters were submitted for the poster session, including submissions from individuals external to the University of Utah, as well as students, staff, and faculty from all areas of campus. Posters were individually advertised on the EHSL Twitter account prior to the conference to increase interest and participation. A team of faculty from EHSL judged the posters and awarded prizes for the top two posters.

Over 200 individuals registered for the conference. Registrants came from nearly all disciplines, colleges, and schools on campus, even though the speakers slanted toward a biomedical focus. Of particular note was the interest from faculty in philosophy, psychology, engineering, computer science, social work, law, and education, all disciplines that EHSL normally does not serve. Attendees from other institutions also participated, including medical librarians and medical faculty from across the nation.

To further increase the reach of the conference, we livestreamed it on YouTube—where we had over 100 viewers locally, nationally, and internationally during the conference—and posted the individual sessions, if permissions were granted, on YouTube post-conference [25]. Remote viewers were also encouraged to participate on Twitter using the conference hashtag.

Because we used Twitter for promotion, our campus’s science marketing team picked up on the conference early. This was highly fortuitous for us, because they asked if they could bring in Christie Aschwanden, noted science journalist for FiveThirtyEight, as an additional speaker. We worked with them to add Aschwanden for a special lunchtime seminar in between the Center for Open Science post-conference workshops. No registration was required to attend her lecture, and over 150 students, staff, and faculty attended, so the room was over capacity. The early connection with marketing also brought us additional promotion through official University of Utah Twitter channels and podcasts on *The Scope,* the University of Utah Health radio station [26, 27].

After the conference, we sent attendees a follow-up survey to determine their satisfaction with the conference. Those seeking CME credit were required to complete an additional evaluation tool to receive credit. We gathered forty-seven responses from the primary post-conference survey. Of those that responded, forty-five rated the conference as either excellent or good, and forty-four said they were very likely or likely to attend a future research reproducibility conference. Similar positive responses were given for each conference speaker and event. Participants commented on the value of the conference’s networking opportunities, the superb quality of the speakers, and the necessity of continuing the conversation. One respondent said, “You’ve organized this terrific event. Please repeat. I mean it.” Results from fourteen respondents to the CME evaluation provided similar themes.

We also asked for qualitative feedback regarding participants’ major takeaways from the events and what they would like to see at future events. Takeaways primarily centered on newfound awareness of the problem and specific tools or readings to investigate, but a few respondents were disappointed by the attendance. One respondent noted the takeaway “[t]hat apparently no one at Utah has a research reliability problem since so few attended. Quite disheartening since the problems here are substantial.” For future events, respondents asked for more opportunities to network and partner on research throughout the year, practical and realistic applications and tools for solving the problem, follow-up sessions and talks, and movement toward “meaningful action and change.” One respondent also suggested that the “[VPR] ought to make such attendance mandatory to qualify for [a] grant award on campus.”

After the conference, we collaborated with the VPR’s office to lead discussions on how we might move forward as an institution. We suggested key faculty attendees for the first discussion, led by the associate VPR. This collaboration provoked excellent discussion of concrete ideas, including building a weeklong, credit-bearing course into the next conference; creating a group for on-campus journal editors; and networking across disciplines. In addition, one of the authors (Rethlefsen) led a follow-up discussion with the research deans group to get additional feedback and gauge interest in educational or training sessions for their faculty and students.

Going forward, we are using feedback from the conference and discussion sessions to create a plan for future directions. We are currently planning a second conference for 2018 that will kick off a weeklong credit- and certificate-bearing course. We have partnered with the Department of Philosophy to offer credit for the course, and we have already received commitments for sponsorships from the VPR and CCTS for the conference. The CCTS will also ask their trainees to attend the course, ensuring at least twelve students for the weeklong session.

We are working with the VPR to investigate making this training mandatory for grantees on campus. In addition, to retain momentum, we are starting research reproducibility grand rounds in September 2017. This yearlong program will enable reproducibility-engaged students, staff, and faculty to hear from experts across campus on how their disciplines ensure reproducibility. To complement the grand rounds sessions, we are partnering with College of Nursing faculty and the Research Administration Training Series (RATS) director to plan practical courses to teach reproducibility skills.

Lastly, we formed a Research Reproducibility Coalition, which will meet quarterly, to enable members to network and keep other groups on campus informed about work going on in their areas. The Research Reproducibility Coalition will also help provide guidance on curriculum development and conference programming.

We continue to use the #MakeResearchTrue hashtag to promote reproducibility resources and guidance, and we have been encouraging others to adopt this hashtag. We also use the more widely known #reproducibility and #openscience hashtags to draw in additional researchers. One member of our team partnered with another medical librarian colleague to develop the “Librarian’s Role in Reproducibility of Research Symposium,” which was offered at MLA ‘17 [28]. Another member of our team will teach a two-and-a-half-day short course on “Principles and Practices for Reproducible Science” through the DeCART summer program, a new training opportunity offered through the university’s Department of Biomedical Informatics [29]. To increase our capacity, we added reproducibility and open science as a major component of a job position we created in 2017.

## DISCUSSION

Research reproducibility continues to be a major topic of discussion amongst researchers, funders, and the public [30]. Health sciences librarians are well positioned to help lead the discussion on their campuses and more broadly. At our institution, we have been very fortunate to build strong alliances and partnerships with groups on campus, particularly the VPR office, who have not only supported and encouraged us, but who have also helped us financially and, most critically, by using their influence to make reproducibility a campus goal. Other partnerships with marketing, the CCTS, RATS, and faculty and staff in the various schools and colleges enabled us to promote and sustain our efforts.

Sustainability of our efforts not only requires partnerships, but also substantial investment and commitment amongst our library faculty. Three faculty currently work on this topic, and many more will be drawn in to speak at our research reproducibility grand rounds or teach future courses. Sustainability also requires substantial financial investment. Our 2016 conference cost nearly $20,000 to execute, even with many speakers refusing or unable to accept honoraria or, in some cases, even travel costs.

We have already secured two sponsors for next year’s conference: our CCTS and VPR. We have also sought grant funding from national and regional sources to sustain these efforts, and we intend to continue funding at least one to two lecturers from library endowments. Lastly, we intend to charge for attendance at future large events, at least a minimal fee, to increase attendance and commitment amongst those who registered.

Though this effort has required major investments of time and money, the enormously positive reaction and encouragement that we have received from across campus and even externally has more than justified our costs. Subsequent to the conference, campus groups have approached us to form partnerships, and many of these groups have committed money, faculty time, and even grant coinvestigator status to our team members due to our work. Our Research Reproducibility Coalition, which we announced via email to interested faculty, was received enthusiastically, with many faculty responding within minutes.

Even though we have a long way to go, we believe our case study clearly demonstrates the ability of the library to directly and broadly impact how reproducibility is addressed and achieved [31]. Though we committed funds for programming, many of our most critical program components—such as connecting with our VPR office, conducting social media outreach, bringing together local journal editors and experts for panels and discussion, and creating our upcoming research reproducibility grand rounds series—cost only time and could be components of any library’s outreach efforts. We encourage other health sciences librarians to engage in this highly important area.
